# Highly Efficient and Stable Organic Light‐Emitting Diodes with Inner Passivating Hole‐Transfer Interlayers of Poly(amic acid)‐Polyimide Copolymer

**DOI:** 10.1002/advs.202105851

**Published:** 2022-01-27

**Authors:** Jaewoo Park, Wonsun Kim, Yushika Aggawal, Kichul Shin, Eun Ha Choi, Byoungchoo Park

**Affiliations:** ^1^ Department of Electrical and Biological Physics Kwangwoon University Wolgye‐Dong Seoul 01897 South Korea; ^2^ Department of Plasma‐Bio Display Kwangwoon University Wolgye‐Dong Seoul 01897 South Korea

**Keywords:** hole‐transfer, inner encapsulation, interlayer, lifetime, organic light‐emitting devices, poly(amic acid)‐polyimide copolymer, stability

## Abstract

Ensuring the long‐term stability of high‐performance organic light‐emitting diodes (OLEDs) has remained a great challenge due to their limited lifetime and durability. Herein, a novel functional interlayer consisting of a poly(amic acid)‐polyimide copolymer is introduced for use in OLEDs. It is shown that an OLED sample with a polyimide‐copolymer interlayer exhibits high peak brightness of nearly 96 000 cd m^−2^ and efficiency of ≈92 cd A^−1^, much higher than those (≈73 000 cd m^−2^ and ≈83 cd A^−1^) of a well‐organized reference OLED. Moreover, the growth of dark spots is strongly suppressed in the sample OLED and the device lifetime is extended considerably. Further, highly stable and uniform large‐area OLEDs are successfully produced when using the interlayer. These improvements are ascribed not only to the excellent film‐forming and hole‐transferring properties but also to the inner passivating capability of the polyimide‐copolymer interlayer. The results here suggest that the introduction of an inner passivating/encapsulating hole‐transferable polyimide‐copolymer interlayer together with conventional external encapsulation technology represents a promising breakthrough that enhances the longevity of high‐performance next‐generation OLEDs.

## Graphical abstract

An interlayer composed of polyimide copolymer is introduced in organic light‐emitting diodes (OLEDs) as an inner passivating charge‐selective interlayer. Due to its good film‐forming ability, excellent hole‐transferring (and electron‐blocking) properties, and effective permeation‐inhibiting properties with regard to water molecules, bright, efficient, and robust OLEDs are successfully realized together with strong suppression of the growth of dark spots in the OLEDs.

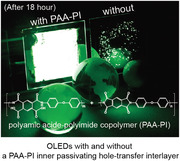

## 1 Introduction

Numerous studies have recently focused on the development of organic, polymeric, and organic/inorganic semiconducting functional materials and related device structures for use in light‐emitting diodes (LEDs) in an effort to implement high‐end applications of full‐color flat‐panel information displays and solid‐state lighting.^[^ ^1^, ^2^, ^3^, ^4^, ^5^, ^6^, ^7^ ^]^ Examples include organic, polymer, and/or perovskite LEDs (OLEDs, PLEDs, and/or PeLEDs). In order to achieve these challenging goals, studies have focused on improving device performance and stability and on the simplicity of the device fabrication process for cost‐efficient, lightweight, flexible, and large‐area LEDs, especially OLEDs.^[^ ^8^, ^9^, ^10^ ^]^ For example, with respect to the device performance of OLEDs, researchers have achieved internal quantum efficiencies approaching 100% by incorporating phosphorescent^[^ ^11^, ^12^, ^13^, ^14^ ^]^ or thermally activated delayed fluorescent^[^ ^15^, ^16^, ^17^ ^]^ guest emitters dispersed into the host materials of emitting material layers (EMLs), leading to efficient radiative transitions through an (reverse) intersystem crossing from triplet states to a ground state.^[^ ^2^, ^17^, ^18^ ^]^ The use of these types of materials has enabled the realization of OLEDs with a high peak luminescence level approaching the range of 50 000–100 000 cd m^−2^ and peak efficiency exceeding 25–60 cd A^−1^.^[^ ^2^, ^17^ ^]^ As another example, related to the simplicity of the device fabrication process, solution‐processable OLEDs, such as slot‐die‐coated, inkjet‐printed, bar‐coated, and roll‐to‐roll‐coated OLEDs, have also been gradually developed.^[^ ^19^, ^20^, ^21^, ^22^, ^23^ ^]^ However, current OLEDs continue to be associated with several serious drawbacks, such as poor device stability levels and the expectation of short lifetimes, given their extreme sensitivity to oxygen and moisture, which cause an aggravation in the degradation process in these devices.^[^ ^8^, ^24^, ^25^ ^]^

The extreme sensitivity of OLED materials and devices to moisture and oxygen demands highly controlled production environments together with defect‐free encapsulation technologies, including single‐barrier thin‐film encapsulation (TFE) methods such as atomic layer deposition^[^ ^26^, ^27^, ^28^, ^29^, ^30^ ^]^ or chemical vapor deposition (CVD)^[^ ^30^, ^31^, ^32^ ^]^ as well as multibarrier TFE methods that entail the repeated stacking of organic/polymeric and inorganic layers.^[^ ^9^, ^33^, ^34^, ^35^ ^]^ Nevertheless, such TFE methods, which are mainly associated with protection against oxidation and delamination of the metal cathode at the cathode/organic interface, have led to complicated fabrication processes and thus increased production costs. In particular, TFE technologies are linked to the disadvantages of both limited processing conditions and limited material selection, together with potential damage to devices through the direct deposition process.^[^ ^36^ ^]^ Hence, the conventional external TFE methods, related processes, and materials introduced thus far remain insufficient with regard to protecting OLEDs from degradation. Thus, further progress is required to improve the stability of the OLED itself ultimately to realize mainstream applications in flat‐panel displays and solid‐state lighting.

Apart from the improvements of TFEs mentioned above, another approach to increase the stability of OLEDs is to insert a functional layer into the device structure. For example, an ultrathin layer of poly(paraxylylene) or parylene, prepared by CVD on an indium tin oxide (ITO) anode, could reduce the formation of local nonemissive areas, known as dark spots, by covering the needle‐like defects that can exist in the ITO film, thus extending the device lifetime while also improving device performance.^[^ ^37^ ^]^ However, to ensure the effectiveness of the parylene layer, one must be careful when introducing such a parylene layer so as not to deteriorate the device performance due to difficulties with precise control of the thin film formation process as well as the energy‐level alignment between the adjacent functional layers.

Polyimide (PI), widely used as an excellent dielectric, is a particularly stable polymer consisting of imide monomers that exhibits high chemical durability and good heat resistance. Due to its various advantages strongly depending on its functional groups and thickness,^[^ ^38^, ^39^, ^40^ ^]^ PI is used in a wide range of applications, such as liquid‐crystal (LC) displays, high‐temperature fuel cells, and various other electronic devices.^[^ ^9^, ^41^, ^42^, ^43^, ^44^, ^45^ ^]^ Moreover, owing to its low water vapor transmission rate (≈0.4–21 g m^–2^ d^–1^),^[^ ^41^, ^46^, ^47^ ^]^ PI has recently been used as a substrate for flexible OLEDs.^[^ ^41^, ^47^, ^48^ ^]^ While PI may be an excellent candidate material for versatile functional layers in OLEDs, there has been no systematic study of functional PI layers in OLEDs, possibly because these potential benefits have been overshadowed by the typical insulating properties of conventional thick PI films.^[^ ^45^ ^]^ The few studies available show that thin PI films can be used in OLEDs as alignment layers of LC polymer‐based EMLs for the emission of polarized electroluminescent (EL) light.^[^ ^49^, ^50^ ^]^ Other important roles of thin PI layers, such as charge‐carrier selecting and/or inner passivating layers, have yet to be investigated.

In this study, we report an inner passivating/encapsulating interlayer consisting of functional PI copolymer for high‐performance and robust OLEDs. The functional interlayer used here consists of a random copolymer of poly(amic acid) (PAA, a precursor of PI) and PI (PAA‐PI). PAA‐PI is produced by the thermal imidization of the solution‐processable PAA of poly(pyromellitic dianhydride‐*co*‐4,4′‐oxydianiline) (PMDA‐ODA), which was recently developed as an insulating dielectric possessing a high chain‐packing density for high‐performance organic thin‐film transistors.^[^ ^45^ ^]^ By employing the thin interlayer of PAA‐PI copolymer in the OLED (the sample OLED here), the device not only exhibits excellent device performance much higher than that of a well‐optimized reference OLED but also shows an increased lifetime accompanied by a significant reduction of the growth of dark spots. This level of device performance coupled with the prolonged lifespan are attributed to the characteristics of the functional PAA‐PI interlayer, such as the good match of the energy levels between adjacent layers, the good hole‐transferring, and electron‐blocking properties, and the effective permeation‐ and diffusion‐inhibiting properties with regard to H_2_O molecules. We also assess the feasibility of the device fabrication process for large‐area and high‐performance OLEDs with PAA‐PI interlayers. We therefore demonstrate the effectiveness of the thin PAA‐PI film as an inner passivating/encapsulating hole‐transfer interlayer in the OLED structure, which can be simply extended for use in high‐throughput manufacturing processes.

## 2 Results and Discussion

### 2.1 Characteristics of PAA‐PI Copolymer Thin Films

**Figure 1:**
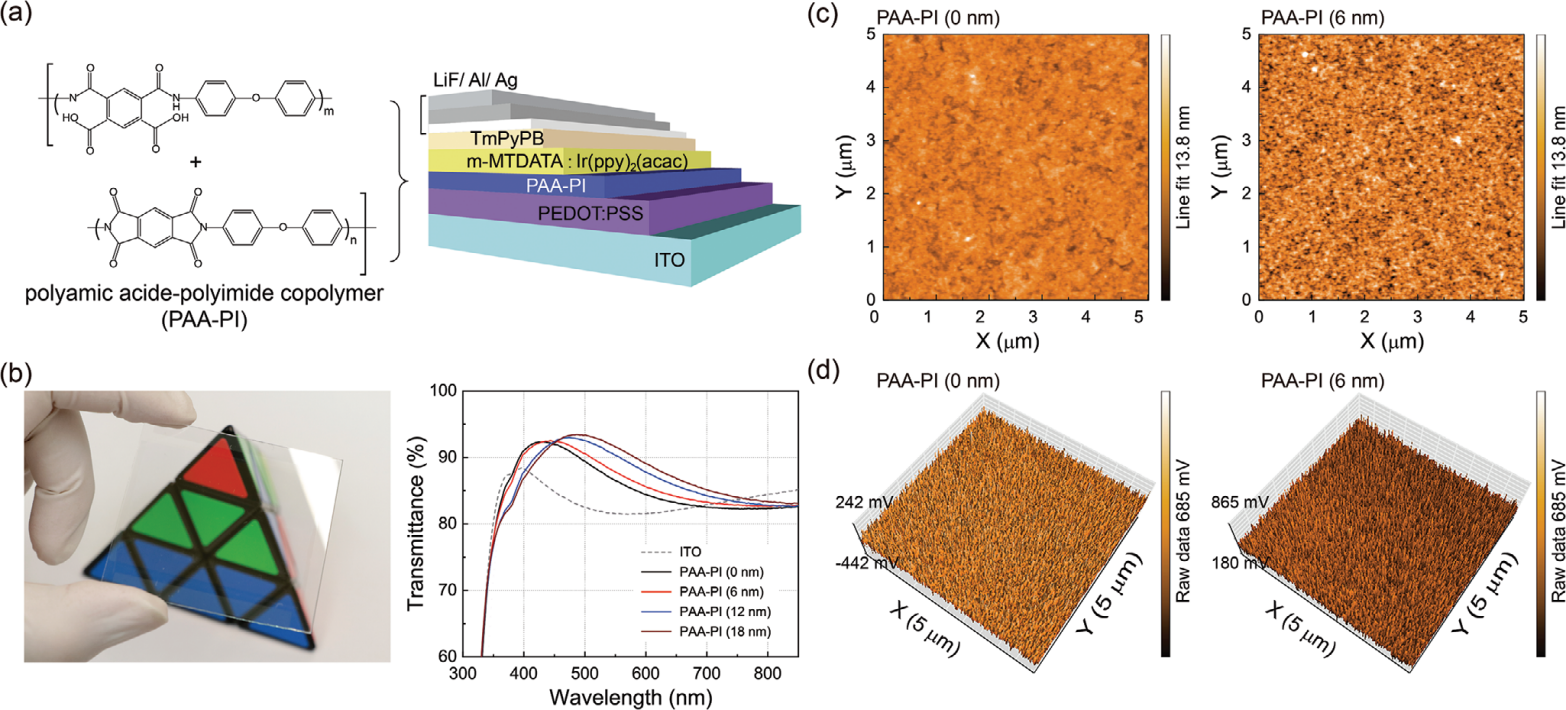
a) Left: Illustration of the molecular structure of the poly(amic acid)‐polyimide copolymer (PAA‐PI) of PMDA‐ODA. Right: A schematic illustration of the OLED structure with a PAA‐PI interlayer. b) Left: Photograph of ITO/PEDOT:PSS/PAA‐PI (6 nm) layers on a 5.5 × 5.5 cm^2^ substrate. Right: UV–vis optical transmission spectra of the ITO/PEDOT:PSS/PAA‐PI layers for several different PAA‐PI film thicknesses. c) AFM topography images and d) corresponding 3D KPFM potential maps of the ITO/PEDOT:PSS/0 nm thick (left) or 6 nm thick (right) PAA‐PI layers.

The chemical structure of the aromatic copolymer of PAA‐PI used for the interlayer is illustrated in **Figure** **1**a, of which the monomer unit is composed partly of PAA, synthesized by polymerizing PMDA with carbonyl chains and ODA having nitrogen atoms, and partly of imidized PI. To form a thin and homogeneous PAA‐PI interlayer, a precursor solution of PAA was spin‐coated onto a hole‐injection layer (HIL) of poly(3,4‐ethylenedioxythiophene):poly(4‐styrenesulphonate) (PEDOT:PSS) and annealed to imidize the PAA layer to the PAA‐PI layer at 180 °C (imidization degree: ≈46%),^[^ ^45^ ^]^ following the procedure described as the synthetic route to the PAA‐PI copolymer (see Figure S1, Supporting Information). Figure 1a also presents a schematic illustration of the OLED structure^[^ ^51^ ^]^ together with an EML of a 4,4’,4”‐tris[phenyl(*m*‐tolyl)amino] triphenylamine (*m*‐MTDATA) host doped with a bis(2‐phenylpyridine) (acetylacetonate) iridium(III) (Ir(ppy)_2_(acac)) guest emitter (m‐MTDATA:Ir(ppy)_2_(acac)), an electron‐transport layer (ETL) of 1,3,5‐tri[(3‐pyridyl)‐phen‐3‐yl]benzene (TmPyPB), a LiF electron‐injecting layer (EIL), and an Al/Ag cathode on the PAA‐PI interlayer.

Prior to the investigation of the effects of the PAA‐PI interlayer on the device performance of the OLEDs, we observed the characteristics of the fabricated PAA‐PI layers. Initially, optical images and the transmission spectra of the PAA‐PI layers on ITO/PEDOT:PSS substrates were characterized (Figure 1b). As shown in the figure, the PAA‐PI copolymer‐coated substrate is highly transparent; as the film thickness of the PAA‐PI layer increases, the average optical transmission level in the visible wavelength range (400–700 nm) increases slightly as the peak position of the transmission spectra of the layers slightly shifts toward the visible wavelength region due to the effect of construct interference in this wavelength region. For example, the average transmission levels in the visible wavelength range for the ITO/PEDOT:PSS/PAA‐PI (6 nm) layers are ≈88% and 89% for the ITO/PEDOT:PSS/PAA‐PI (12 nm) layers and 90% for the ITO/PEDOT:PSS/PAA‐PI (18 nm) layers, all of which are comparable to that (87%) of the reference of ITO/PEDOT:PSS layer. Hence, PAA‐PI layers on ITO/PEDOT:PSS substrates maintain excellent optical transparency even when introducing a PAA‐PI interlayer.

Subsequently, in order to understand the surface properties of the fabricated PAA‐PI layers, we studied the atomic force microscopy (AFM) topographies of thin PAA‐PI layers on ITO/PEDOT:PSS layers, as shown in Figure 1c (see also Figure S1, Supporting Information). The measured surface roughness value of the reference ITO/PEDOT:PSS layer is ≈0.80 nm, while the corresponding values are ≈1.54, 1.52, and 1.27 nm for the 6, 12, and 18 nm thick PAA‐PI layers on the ITO/PEDOT:PSS layers, respectively. Thus, it is apparent that the PAA‐PI layers formed on the PEDOT:PSS layers exhibit fairly smooth and homogeneous surfaces without any needle‐like defects or pinholes in the coated layers. For comparison, we also observed the Kelvin probe force microscopy (KPFM) surface potentials^[^ ^52^ ^]^ of the PAA‐PI layers. As shown in Figure 1d, the obtained KPFM surface potential maps confirm the uniform and homogeneous surfaces of the PAA‐PI layers. With these KPFM surface potential maps, the contact potential differences (CPDs) of the PAA‐PI layers were also determined; these are ≈−84 mV for the reference ITO/PEDOT:PSS layer, 335 mV for the 6 nm thick PAA‐PI layer, 501 mV for the 12 nm thick PAA‐PI layer, and ≈554 mV for the 18 nm thick PAA‐PI layer on ITO/PEDOT:PSS. Thus, it is clear that the surface potential begins to increase as the film thickness of the PAA‐PI layer increases. Based on these surface potentials, it is possible to deduce the dipolar orientational information of the PAA‐PI, possessing strong polar carboxyl and amide groups (—COOH/—CONH);^[^ ^41^, ^44^, ^45^, ^46^ ^]^ the dipole moments of PAA‐PI in the layer appear to be directed away from the underlying PEDOT:PSS layer, possibly causing increases in the CPDs.

**Figure 2:**
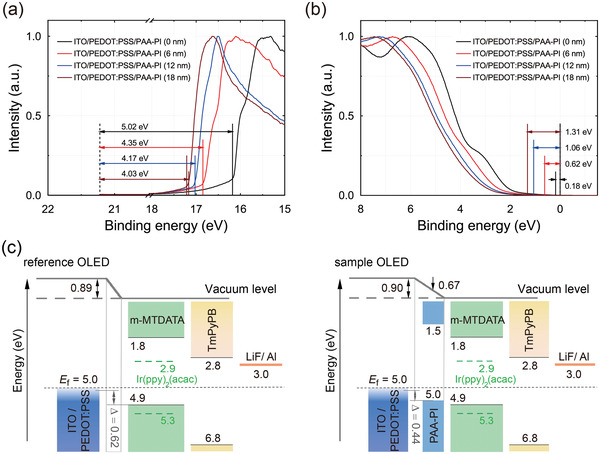
a) Ultraviolet photoelectron spectroscopy (UPS) spectra of thin PAA‐PI layers for several different film thicknesses on ITO/PEDOT:PSS layers for the determination of their work functions and b) valence band maximum values. c) Energy‐level diagrams of OLEDs without (reference, left) and with (sample, right) a PAA‐PI interlayer (6 nm). Energy barriers Δ*s*: Δ_reference_ = Δ*E*_F(PEDOT:PSS/m‐MTDATA)_–Δ*E*_HOMO(PEDOT:PSS/m‐MTDATA)_ = (5.02–4.13)–(5.20–4.93) eV ≈0.62 eV between the PEDOT:PSS HIL and the m‐MTDATA layer for the reference OLED, while Δ_sample_ = Δ*E*_F(PEDOT:PSS/PAA‐PI)_–Δ*E*_HOMO(PEDOT:PSS/PAA‐PI)_ = (5.02–4.35)–(5.20–4.97) eV ≈0.44 eV between the PEDOT:PSS HIL and the PAA‐PI interlayer (6 nm) for the sample OLED.

In order to confirm the aforementioned observations of the surface potentials and to obtain further information pertaining to the thin PAA‐PI layers, we investigated the electronic structure of the PAA‐PI layers by means of ultraviolet photoelectron spectroscopy (UPS). **Figure** **2**a,b shows the observed UPS spectra (He I) of the PAA‐PI layers on ITO/PEDOT:PSS, providing information about the photoemission cutoff energy and the energy difference between the Fermi level (*E*_F_) and the valence band maximum (*E*_VBM_) (or the highest occupied molecular orbital (HOMO) level, *E*_HOMO_), respectively. It is clearly seen from the positions of the photoemission cutoffs that the estimated levels of the work function and *E*_HOMO_ for the reference ITO/PEDOT:PSS layer are ≈5.02 and 5.20 eV, respectively, very close to previously reported UPS measurements.^[^ ^53^ ^]^ Likewise, the estimated work functions for the ITO/PEDOT:PSS/PAA‐PI layers are determined to be ≈4.35 eV for the 6 nm thick PAA‐PI layer, 4.17 eV for the 12 nm thick PAA‐PI layer, and 4.03 eV for the 18 nm thick PAA‐PI layer on ITO/PEDOT:PSS (Figure 2a and **Table** **1**). The clear decrement in the work function of the ITO/PEDOT:PSS/PAA‐PI layers implies that some of the dipole moment of the PAA‐PI is directed away from the PEDOT:PSS layer,^[^ ^54^ ^]^ which confirms the observed results of the KPFM surface potentials, as shown above. Further, the HOMO energy levels, *E*_HOMO_s, of the ITO/PEDOT:PSS/PAA‐PI layers were also estimated using the UPS data (Figure 2b). The estimated *E*_HOMO_s of the layers are determined to be ≈4.97 eV for the 6 nm thick PAA‐PI layer on ITO/PEDOT:PSS, 5.23 eV for the 12 nm thick PAA‐PI layer, and 5.34 eV for the 18 nm thick PAA‐PI layer on ITO/PEDOT:PSS (Table 1). These results clearly indicate that introducing the thin PAA‐PI layer effectively modulates the work function and the *E*_HOMO_ level (4.97–5.31 eV) of the ITO/PEDOT:PSS layers.

**Table 1:** Summary of the energy levels for the functional layers studied here for several different film thicknesses of the PAA‐PI interlayers

Layers [thickness nm]	Work function [eV]	*E*_F_ – *E*_HOMO_ [eV]	*E*_HOMO_ [eV]
ITO	4.51	—	—
ITO/PEDOT:PSS/PAA‐PI (0)	5.02	0.18	5.20
ITO/PEDOT:PSS/PAA‐PI (6)	4.35	0.62	4.97
ITO/PEDOT:PSS/PAA‐PI (12)	4.17	1.06	5.23
ITO/PEDOT:PSS/PAA‐PI (18)	4.03	1.31	5.34
ITO/PEDOT:PSS/PAA‐PI (0)/m‐MTDATA	4.13	0.80	4.93
ITO/PEDOT:PSS/PAA‐PI (6)/m‐MTDATA	4.12	0.81	4.93
ITO/PEDOT:PSS/PAA‐PI (18)/m‐MTDATA	3.98	0.92	4.90

Together with the estimated energy levels of the other functional layers of m‐MTDATA (Table 1 and Figure S2, Supporting Information), we devised energy‐level diagrams of OLEDs without (reference) and with the PAA‐PI interlayer (sample) (Figure 2c). As shown in the diagram on the left in Figure 2c for the reference device, a large difference (≈0.89 eV) in the vacuum‐level positions at the interface region between the PEDOT:PSS HIL and the m‐MTDATA EML is estimated, primarily due to the intrinsic formation of an interface dipole at the HIL/EML interface.^[^ ^54^ ^]^ This diagram also shows a large energetic barrier (Δ≈0.62 eV) between the HOMO levels of the HIL and EML. In contrast, for the sample device, when the PAA‐PI interlayer is introduced between the HIL and EML, the vacuum‐level position at the interface region between the HIL and EML varies as a function of the thickness of the PAA‐PI interlayers (diagram on the right in Figure 2c and Figure S3, Supporting Information). Such clear vacuum‐level variations of ≈0.01–0.15 eV in the diagrams are mainly due to the dipole effect of PAA‐PI in the interlayer, the dipole moment of which points in the opposite direction from the PEDOT:PSS HIL, as mentioned above. Notably, it was also found that for the sample device with the 6 nm thick PAA‐PI interlayer, the HOMO level of the PAA‐PI interlayer is located between the HOMO levels of the HIL and EML, reducing the energy barrier, Δ, to ≈0.44 eV (panel on the right in Figure 2c). However, when the thickness of the PAA‐PI interlayer exceeds 18 nm, the HOMO level of the PAA‐PI interlayer begins to be higher than those of the HIL and EML, increasing Δ to more than ≈1.1 eV, inversely (Figure S3, Supporting Information). Furthermore, the large energy barrier (≈0.9–1.1 eV) between the lowest unoccupied molecular orbital (LUMO) levels of the 6 nm thick PAA‐PI interlayer and the EML, shown in Figure 2c, may permit the PAA‐PI interlayer to act as an electron‐blocking interlayer in the sample device. Thus, the observed physical and electrical properties of the PAA‐PI interlayer can affect the device performance together with the device stability of OLEDs, as investigated and discussed below.

### 2.2 Device Performance of OLEDs with PAA‐PI Interlayers

**Figure 3:**
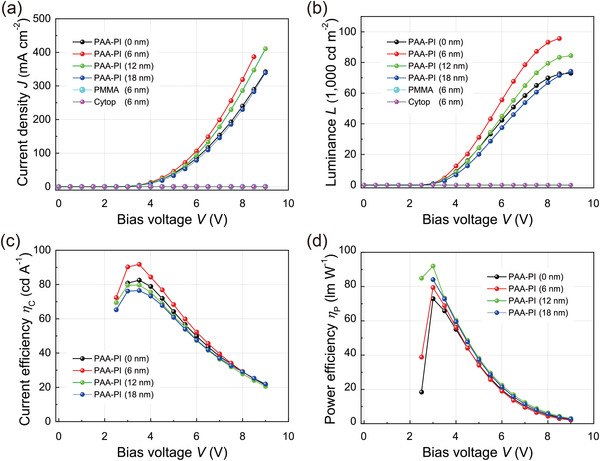
a) Current density–voltage (*J*–*V*), b) luminance–voltage (*L*–*V*), c) current efficiency–voltage (*η*_c_–*V*), and d) power efficiency–voltage (*η*_p_–*V*) characteristics of OLEDs as a function of the film thickness of the PAA‐PI interlayer. For comparison, *J*–*V* and *L*–*V* curves of OLEDs with different interlayers of PMMA (6 nm) or CYTOP (6 nm) are also shown.

In order to investigate the effects of the PAA‐PI interlayer on the EL performances of OLEDs, we fabricated OLEDs, each with a PAA‐PI interlayer (0, 6, 12, or 18 nm, Figure 1a). Here, we describe the device characteristics in terms of the current density (*J*) and luminance (*L*) as a function of the applied voltage (*V*) for the sample OLEDs with the PAA‐PI interlayers, in comparison with a well‐optimized reference OLED^[^ ^51^ ^]^ without an interlayer (0 nm). **Figure** **3**a shows the observed current density–voltage (*J*–*V*) characteristics of the fabricated OLEDs. For comparison, the *J*–*V* characteristics of other OLEDs with different polymeric interlayers of poly(methyl methacrylate) (PMMA) or cyclized transparent optical fluoropolymer (CYTOP) instead of PAA‐PI are also presented in the figure. As shown in the figure, the current densities flowing through the functional layers of the sample OLEDs are much higher than those of the comparative OLEDs with the PMMA or CYTOP interlayer and are similar to that of the reference OLED. This result clearly demonstrates the good charge (hole)‐transfer properties of the thin PAA‐PI interlayer studied here, in contrast to other thin insulating layers of PMMA and CYTOP. It should also be noted that as the film thickness of the PAA‐PI interlayer increases in the sample OLED, its current density begins to decrease slightly.

Subsequently, it is also important to note that the sample OLEDs exhibit significantly high luminance–voltage (*L*–*V*) performance capabilities (Figure 3b). The maximum luminance (*L*) of the sample OLED with the 6 nm thick PAA‐PI interlayer is *L* ≈ 96 000 cd m^–2^ at *V* = 8.5 V with a low threshold voltage (*V*_onset_) of ≈2.5 V. This maximum luminance *L* is higher than that (*L* ≈ 73 000 cd m^–2^ at *V* = 8.5 V, *V*_onset_ of ≈2.5 V) of the reference OLED and is also much higher than those (*L* ≈ 6–20 cd m^–2^ at *V* = 12.0 V, *V*_onset_ of ≈5.0–6.5 V) of the comparative OLEDs with the PMMA or CYTOP interlayer. The current efficiency *η*_c_ and the power efficiency *η*_p_ of the sample OLED are thus remarkably higher than those of the reference OLED, as shown in Figure 3c,d, respectively. For instance, the highest *η*_c_ and *η*_p_ values observed from the sample OLED with the 6 nm thick PAA‐PI interlayer are correspondingly ≈91.8 cd A^–1^ and 79.4 lm W^–1^ (at *V* = 3.5 V), which are much higher than those (82.5 cd A^–1^ and 72.9 lm W^–1^) of the reference OLED. Moreover, even at high luminance levels of 50 000 cd m^–2^, the sample OLED maintains a high *η*_c_ of ≈56.0 cd A^–1^ and *η*_p_ of 22.4 lm W^–1^, which are also significantly higher than those (*η*_c_ = 44.0 cd A^–1^ and *η*_p_ of 15.0 lm W^–1^) of the reference OLED. Further, the highest external quantum efficiency (EQE) value observed from the sample OLED is ≈19.6%, which is also higher than that (18.6%) of the reference OLED. Thus, the observed *J*–*L*–*V* results clearly demonstrate that the thin PAA‐PI interlayer introduced between the HIL and the EML can serve as a good hole‐transfer interlayer, which can therefore improve the excellent device performance capabilities of well‐optimized reference OLEDs even more.^[^ ^51^ ^]^ In addition, introducing the PAA‐PI interlayer between the PEDOT:PSS HIL and the EML can help to prevent radiative exciton quenching (see Figure S4, Supporting Information), which may arise at the interface between the PEDOT:PSS and the emission layer.^[^ ^55^ ^]^ This benefit can also increase the efficiency of the sample OLEDs.

**Figure 4:**
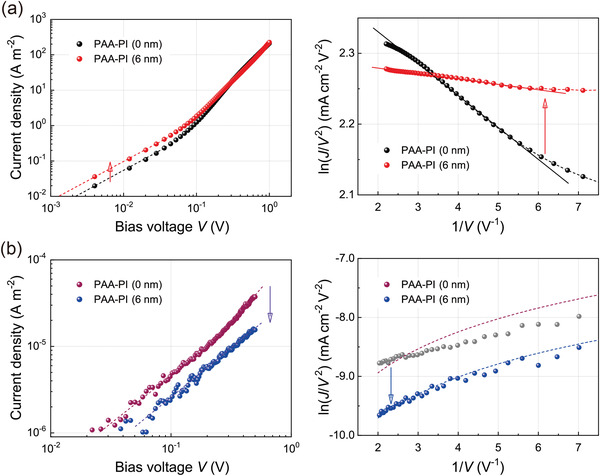
*J*–*V* characteristics of a) hole‐only devices and b) electron‐only devices without (0 nm, reference) and with a PAA‐PI interlayer (6 nm, sample). Left: Ohmic plots on logarithmic scale. Right: F‐N tunneling plots.

Next, to gain a deeper understanding of the effects of the PAA‐PI interlayer on the hole‐current flow characteristics of the sample OLED, hole‐only devices (HODs) without (reference) and with (sample) a 6 nm thick PAA‐PI interlayer were also fabricated and investigated. The *J*–*V* characteristic curves as observed in a darkroom of the fabricated HODs are illustrated in **Figure** **4**a, showing two different regions; when the applied voltage is below ≈0.1 V, the slope of ln(*J*) versus ln(*V*) is 1, which suggests that Ohmic conduction is dominant. When the applied voltage exceeds ≈0.1 V, the flowing current densities can be explained as the space‐charge‐limited current (SCLC) mechanism,^[^ ^56^, ^57^ ^]^ showing a linear relationship with a slope of 2. Thus, the Ohmic and SCLC conduction types can be the main bulk‐limited conduction mechanisms in both HODs (see also Figure S4a, Supporting Information). Hence, the measured current flows for the HODs can be described in terms of the Ohmic (*J*_Ohmic_) and SCLC (*J*_SCLC_) conduction mechanisms using the following equation^[^ ^56^, ^57^ ^]^

(1) $$J\;\left(V\right)=J_{\mathrm{Ohmic}}\;+\;J_{\mathrm{SCLC}}=\frac{ne\mu_{p}}{d}\;V+\frac{9\mu_{p}\theta_{f}\varepsilon_{r}\varepsilon_{0}}{8d^{3}}V^{2}$$

Here, *n* is the concentration of the charge carriers (holes), *e* is the unit charge of 1.6 × 10^–19^ C, and *μ*_p_ is the hole mobility (≈3.0 × 10^–9^ m^2^ V^–1^ s^–1^ for the m‐MTDATA:Ir(ppy)_2_(acac) layer).^[^ ^58^ ^]^ *ε*_r_ and *ε*_0_ represent the relative permittivity (≈3.0)^[^ ^58^ ^]^ of the functional organic materials used here and the vacuum permittivity (8.85 × 10^–12^ F m^–1^), respectively. *θ*_f_ denotes the proportion of the free carrier density (*n*_free_) to the total carrier density (*n*_t_),^[^ ^56^, ^57^ ^]^ which is the sum of the trapped (*n*_trapped_) and free carrier density (*n*_t_ = *n*_free_ + *n*_trapped_). *d* is the effective thickness of the functional layers between the anode and cathode; these values are 45 nm (EML) and 51 nm (PAA‐PI/EML) for the reference and sample HODs, respectively. From the analysis of the *J*–*V* curves with Equation (1) for the HODs, the carrier concentration in the Ohmic region for the sample HOD is estimated to be *n* = 1.1 × 10^15^ cm^–3^, nearly twice that (5.6 × 10^14^ cm^–3^) of the reference HOD. This result indicates that the hole conductivity (*n e μ*_p_ = 6.7 × 10^–7^ m^–1^ Ω^−1^) of the sample HOD is also nearly twice that (3.0 × 10^–7^ m^–1^ Ω^–1^) of the reference HOD. Further, *θ*_f_ in the SCLC region for the HODs can be obtained in each case using Equation (1). The estimated value of *θ*_f_ for the sample HOD is 0.29, fairly large compared to that (0.21) of the reference HOD. Hence, the concentration ratio of trapped and free hole carriers (*n*_trapped_/*n*_free_) for the sample HOD is estimated to be ≈2.4, which is much less than that (≈3.8) of the reference HOD. Thus, the use of PAA‐PI interlayers can lead to a reduction of trapped hole carriers at the interface between the HIL and EML and in turn may facilitate an improvement of the stability of the device.^[^ ^57^ ^]^

The panel on the right in Figure 4a shows Fowler–Nordheim (F‐N) plots with ln(*J*/*V* ^2^) versus *V^−^* ^1^ in order to access the interface‐limited conduction of the HODs.^[^ ^59^, ^60^ ^]^ As shown in the figure, F‐N tunneling occurs when the applied voltage exceeds ≈0.12–0.13 V for both HODs, showing that F‐N tunneling is the main interface‐limited conduction mechanism in both HODs (see also Figure S5a, Supporting Information). From the analysis of the negative slopes of the ln(*J*/*V* ^2^) versus *V* ^−1^ curves,^[^ ^59^, ^60^ ^]^ the potential barrier height (*Φ*) in the F‐N tunneling region for the sample HOD is estimated to be *Φ* = ≈0.06 eV, which is approximately half as high as that (*Φ* = ≈0.10 eV) for the reference HOD. Such a large reduction in *Φ* is clearly attributable to the reduced energy barrier (Δ) of the HOMO levels between the HIL and EML caused by the introduction of the PAA‐PI interlayer, as shown above in the energy‐level diagrams (Figure 2c).

Next, in order to investigate the electron‐blocking capability of the PAA‐PI interlayer, we also assessed the electron‐current flows versus the applied voltage (*J*–*V*) characteristics of fabricated electron‐only devices (EODs). These measurements (panel on the left in Figure 4b) clearly show that the EOD with the PAA‐PI interlayer (sample) exhibited much smaller electron‐current flows through the functional layers compared to the EOD without the interlayer (reference), also governed by the Ohmic and SCLC conduction mechanisms (see Figure S5b, Supporting Information). Moreover, the panel on the right of the F‐N plots shows that the direct tunneling of electrons (dotted curves)^[^ ^60^ ^]^ with considerably reduced leakage current occurs in the sample EOD when introducing the PAA‐PI interlayer (see also Figure S5b, Supporting Information). Thus, the PAA‐PI interlayer introduces efficient electron‐blocking abilities, mainly stemming from the large energy barrier between the LUMO levels of the PAA‐PI interlayer and the m‐MTDATA EML, as described above. This demonstrates that the use of the PAA‐PI interlayer may lead to a reduction of the electron leakage current through the functional layers, likely improving the stability as well as the device performance of the device.^[^ ^61^, ^62^ ^]^

### 2.3 Device Stability of OLEDs with PAA‐PI Interlayers

We subsequently investigated another important functionality of the PAA‐PI interlayer as an inner passivating interlayer considering its excellent thermal and chemical durability.^[^ ^38^, ^39^, ^40^, ^41^, ^42^ ^]^ Herein, the storage stability of the fabricated OLEDs (without any external encapsulation) was initially observed in terms of the current density *J* flowing through devices without (reference) and with (sample) the 6 nm thick PAA‐PI interlayer. Because the storage lifetime is closely related to the formation and expansion of dark spots that occur when OLEDs are in storage,^[^ ^8^ ^]^ observing the *J*–*V* characteristics of stored OLEDs can provide information about the degradation mechanisms in the OLEDs. To measure the storage stability, the fabricated OLEDs were stored in a nitrogen glovebox between successive *J*–*V* curve measurements. The *J*–*V* curve measurements were taken at an applied voltage within the range of 0.0–6.0 V at room temperature, allowing the flowing current densities to be kept below 15 mA cm^–2^ in order to minimize current‐induced degradation of the devices during the measurement process.

**Figure 5:**
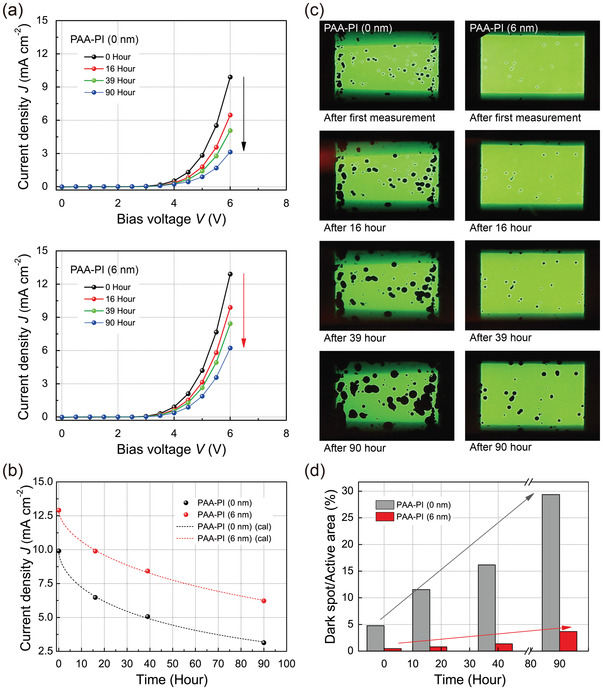
a) Successive *J*–*V* characteristics of a reference OLED (upper) and sample OLED with a 6 nm thick PAA‐PI interlayer (lower) (23 °C, 30% RH, without any external encapsulation) for several different storage times of 0, 16, 39, and 90 h in a N_2_ glovebox. b) Decrements in the current densities of the reference and sample OLEDs at an applied voltage of 6.0 V as a function of the storage time. c) EL light‐emission images of reference (left) and sample (right) OLEDs (at *V* = 4.0 V) taken just after each *J*–*V* measurement as a function of the storage time. d) Area ratios of the dark spots to the light‐emitting active area as a function of the storage time for the reference and sample OLEDs.

**Figure** **5**a shows the *J*–*V* characteristics of two representative OLEDs stored for several different storage times. As shown in the figure, the current density of the reference OLED decreased significantly as the storage time was increased. In contrast, the sample OLED exhibited relatively small decreases even after longer storage times. Figure 5b presents the observed decrements in the current densities at an applied voltage of 6.0 V as a function of the storage time for both OLEDs. As clearly shown in the figure, the sample OLED is much more stable than the reference OLED. The decrements in the current densities in the figure can be analyzed by means of the stretched‐exponential decay function^[^ ^63^ ^]^

(2) $$J=J_{0}\exp\left[-{\left(t/\tau \right)}^{\beta }\right]$$

where *τ* and *β* are the characteristic time and exponent, respectively. With best‐fit parameters, the theoretical curves from Equation (2) are also shown in the figure as dotted curves. The estimated *τ* and *β* values of the reference OLED are 73.3 h and 0.58, respectively. In contrast, notably, the estimated parameter values of the sample OLED are *τ* = 155.6 h and *β* = 0.59. This analysis shows that the PAA‐PI interlayer significantly promotes storage stability in the sample OLED with a 2.2‐fold increase in the characteristic time *τ* with respect to that of the reference OLED.

Hence, in order to understand the passivation effect of the PAA‐PI interlayer in the sample OLED, we also observed the EL light emission from the OLEDs studied here. Figure 5c shows a series of EL light‐emission images of reference and sample OLEDs (at *V* = 4.0 V) taken just after each *J*–*V* measurement (Figure 5a) as a function of the storage time. As shown in the figure, the dark spots that formed on the reference OLED grew and significantly increased over time. This observation verifies that dark spots formed and grew with the pinhole‐like defects at their centers that create pathways for water and/or oxygen permeation in the functional layers. However, impressively, the growth of the dark spots on the sample OLED was suppressed significantly even without any external encapsulation (Figure 5c,d). Similar results of the remarkably suppressed growth of the dark spots were also observed from the sample OLEDs with the 12 and 18 nm thick PAA‐PI functional interlayers, despite their slightly reduced luminance performance (Figure 3b). Such suppressed growth of these types of dark spots combined with the prolonged storage stability of the sample OLEDs appear to have been primarily caused by the low gas permeability and diffusion of PAA‐PI,^[^ ^41^, ^47^ ^]^ preventing the production of pinhole‐like pathways for water and/or oxygen permeation in the functional layers. Especially with regard to water vapor molecules, due to the strong hydrogen bonding between the penetrating water molecules and the strong polar carboxyl/amide groups (─COOH/─CONH) in PAA‐PI,^[^ ^44^, ^46^ ^]^ the PAA‐PI interlayer in the sample OLED can effectively block any ambient or residual moisture in the device.

**Figure 6:**
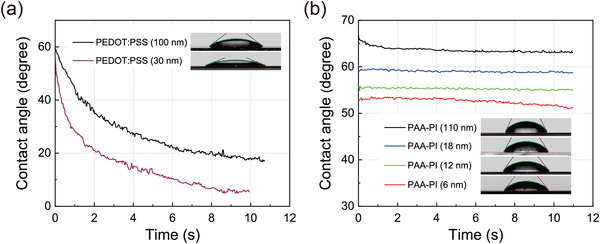
Changes in the contact angles of deionized water droplets on the PEDOT:PSS and PAA‐PI interlayers studied as a function of contact time. a) Water contact angles on the thin (30 nm, gray) and thick (≈100 nm, black) reference PEDOT:PSS layers. b) Water contact angles on the 6 (red), 12 (green), and 18 nm thick (blue) PAA‐PI interlayers on PEDOT:PSS layers (30 nm). The brown curve shows the water contact angle on a thick (110 nm) PAA‐PI layer for comparison. The insets show photographs of water droplets on the functional layers investigated here when contact time = 2 s.

Herein, the interactions between the water molecules and the PAA‐PI interlayers are simply assessed by measuring the water contact angles of the PAA‐PI interlayers. **Figure** **6** shows the measured contact angles of the water droplets as the droplets spread over the studied layers. As shown in Figure 6a, the water contact angles for the thin (30 nm) and thick (≈100 nm) reference PEDOT:PSS layers were in the range of ≈55°–60° when the water droplets initially landed on the PEDOT:PSS surfaces, after which they began rapidly to decrease as the droplets spread. In the later stages of wetting, the contact angles significantly decreased to ≈5° and ≈17° for the thin (30 nm) and thick (≈100 nm) reference PEDOT:PSS layers, respectively, confirming the hydrophilicity of these layers. However, this result also shows the low stability of the PEDOT:PSS layer due to the serious dissolution of the PEDOT:PSS layer arising when the water droplet permeated the PEDOT:PSS layer. In contrast, as shown in Figure 6b, the observed water contact angles of the 6, 12, and 18 nm thick PAA‐PI interlayers on the 30 nm thick PEDOT:PSS layers and that on a 100 nm thick PAA‐PI layer were ≈53°, 56°, 59°, and 67°, respectively, when the water droplets initially landed on the PAA‐PI surfaces. These contact angles also indicate the strong hydrophilic interactions of the PAA‐PI interlayers with the water molecules. Moreover, interestingly, the water contact angles for the thin PAA‐PI interlayers remained mostly unchanged as the contact time increased; even for the thinnest 6 nm thick PAA‐PI interlayer, the decrement of the water contact angle was less than 2° in the later stages. These observations indicate that the PAA‐PI interlayer is quite stable against water permeation and thus that the hydrophilic PAA‐PI interlayer can act as an excellent internal passivation layer. Hence, moisture‐assisted decohesion of the functional layers in an OLED, especially for one with an adjacent HIL and EML, can be efficiently inhibited by the PAA‐PI interlayer, leading to significant reductions in the dark spot growth and thus improving the stability of the device.

**Figure 7:**
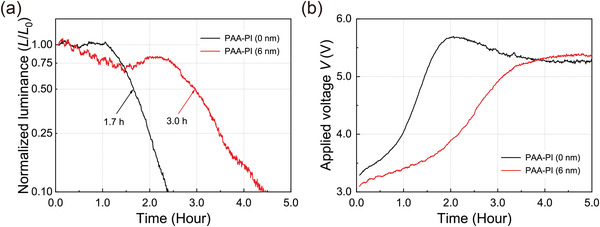
Representative a) luminance lifetime test curves (*L*/*L*_0_–*T*) and b) applied voltage curves of OLEDs without (0 nm, reference) and with a PAA‐PI interlayer (6 nm, sample) under continuous operation in an air atmosphere (23 °C, 30% RH, without any external encapsulation). Each device was driven under a constant current bias to achieve an initial luminance (*L*_0_) of 100 cd m^–2^.

Next, to investigate the driving lifetime, we continuously measured the luminance levels and the driving voltages of the OLEDs studied here as a function of the operating time (*L*/*L*_0_–*T*) under a constant current bias at an initial luminance (*L*_0_) level of 100 cd m^–2^ in air (23 °C, 30% RH, without any external encapsulation) (**Figure** **7**). Here, the operational lifetime (*LT*_50_) of the OLED is referred to as the time for the luminance to decrease to half of the initial value (*L*_0_). As shown in the figure, the luminance lifetime curve of the reference device shows a typical rapid decay outcome. In contrast, interestingly, the sample device offers a clearly longer operational lifetime *LT*_50_ of ≈3.0 h compared to that (≈1.7 h) of the reference device, representing an increase by ≈1.8 times comparing to the reference device. The main reasons for the clearly improved stability of the sample device are summarized below. First, the unique and strong hydrogen bonding of the PAA‐PI interlayer as described above effectively limits the moisture‐assisted decohesion behavior. Second, the reduced the energy barrier between the HOMO levels due to the introduction of the PAA‐PI interlayer effectively prevents excessive hole accumulation at the interface between the HIL and the EML, improving the stability of the sample device, as also described above. In addition, the acid‐induced degradation of the performance of the EML over time can also be effectively prevented by the interlayer of chemically stable PAA‐PI by blocking the sulfonate ions of the PEDOT:PSS HIL.^[^ ^64^ ^]^ Therefore, these results show that an OLED with an interlayer of PAA‐PI as an inner‐passivating hole‐transfer layer can serve as an excellent sustainable alternative to the conventional, more sensitive OLEDs.

### 2.4 Large‐Area OLEDs with PAA‐PI Interlayers

**Figure 8:**
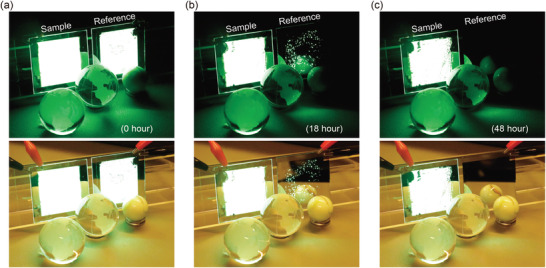
Photographs taken in dark (upper) and bright (lower) ambient light conditions when operating large‐area OLED lighting devices (5.5 × 5.5 cm^2^, at 6.0 V) without (right, reference) and with (left, sample) a solution‐processed 6 nm thick PAA‐PI interlayer a) just after device fabrication, b) after 18 h, and c) after 48 h of storage time in an air atmosphere (23 °C, 30% RH, without any external encapsulation), demonstrating the ease of fabrication of the large‐area and stable OLED with the PAA‐PI interlayer.

Finally, encouraged by the impressive results of the OLEDs with the PAA‐PI interlayer, we assembled large‐area OLEDs without (reference) and with (sample) the PAA‐PI interlayer on 5.5 cm × 5.5 cm ITO‐coated glass substrates to assess the processability of the PAA‐PI thin layer in an OLED. The PAA‐PI interlayer was deposited onto the PEDOT:PSS HIL by means of spin‐coating to produce OLEDs with a light‐emitting pixel size of about 4.0 cm × 5.0 cm. Photographic images of the fabricated devices are shown in **Figure** **8** for several different storage times in air (23 °C, 30% RH, without any external encapsulation). Although the solution‐processed PAA‐PI interlayer was fully fabricated in air, Figure 8a clearly shows that the entire surface of the sample OLED emitted luminous EL flux, similar to that of the reference device. Moreover, the low variation in the EL intensity in the emission area implies low variability with the thickness of the solution‐coated PAA‐PI interlayer. Figure 8b,c shows other EL light‐emission images of the operating sample and of reference OLEDs taken after 18 and 48 h of storage time in an air atmosphere, respectively. A fascinating result here is that after 48 h in air, the sample OLED still emitted relatively bright and homogeneous EL light over the entire active area with very few dark spots, even without any external encapsulation, in contrast to the completely degraded and thus nonemissive reference OLED. Furthermore, the sample OLED emitted EL light even after 186 h (see Figure S6, Supporting Information). These observations clearly demonstrate that the introduction of PAA‐PI interlayers offers the possibility of simple fabrication for bright and stable OLEDs with easy upscaling to larger sizes through a simple solution‐coating process.

All of the results described thus far provide unambiguous evidence that the inner‐passivating hole‐transferable interlayer of PAA‐PI shows considerable promise for the fabrication of high‐performance, stable, and large‐area OLEDs with high throughput and a scalable manufacturing process. It is noteworthy that the device performance of OLEDs with the PAA‐PI interlayers can be improved further by selecting optimal and stable functional materials and/or by combining the device structures with additional functional layers such as hole‐blocking or electron‐transport layers, as reported in the literature. Furthermore, the inner‐passivating hole‐transfer interlayers of PAA‐PI can also be applied to PLEDs, PeLEDs, and other various organic or hybrid (opto)electronic devices together with conventional external encapsulation technologies.

## 3 Conclusion

In summary, we have reported the successful fabrication of highly bright, efficient, stable, and large‐area OLEDs by incorporating solution‐processable hole‐transfer interlayers of PAA‐PI. Homogeneous and smooth PAA‐PI interlayers with controllable thicknesses can be simply deposited from a PAA precursor solution of PMDA‐ODA. It is shown that an OLED with the thin hole‐transfer PAA‐PI interlayer as introduced here exhibited excellent device performance far exceeding that observed from a well‐optimized reference OLED. Moreover, the PAA‐PI interlayer also acted as an inner‐passivating layer in the OLED, resulting in an improvement of the device durability and lifetime together with significant reductions of the dark spot growth phenomenon. We ascribe these improvements to its good film‐forming ability, excellent hole‐transferring (and electron‐blocking) properties, suitable matching of the energy levels between the adjacent functional layers, and effective permeation‐ and diffusion‐inhibiting properties with regard to water molecules in the device. Further, large‐area OLEDs with high uniformity were successfully fabricated using the solution‐processable PAA‐PI interlayers. These results clearly demonstrate that the use of a uniform thin PAA‐PI film as an inner‐passivating hole‐transfer interlayer provides a solid foundation for the further development of high‐performance, stable, and large‐area OLED devices. Furthermore, together with conventional external encapsulation methods, this innovative inner‐passivating hole‐transfer PAA‐PI interlayer can be used in the mass production of high‐performance and stable organic/hybrid lighting and display devices in the near future.

## 4 Experimental Section

### Materials

Bis(2‐phenylpyridine)(acetylacetonate)iridium (III) (Ir(ppy)_2_(acac), C_27_H_23_IrN_2_O_2_), 1,3,5‐tri(3‐pyridyl‐3‐phenyl)benzene (TmPyPB, C_39_H_27_N_3_), and 4,4’,4”‐tris[(3‐methylphenyl) phenylamino] triphenylamine (m‐MTDATA, C_57_H_48_N_4_) were purchased from Lumtec. The PEDOT:PSS aqueous solution used here (Clevios P‐VP‐AI‐4083) was purchased from H.C. Starck. The electron‐injecting material of LiF and the PAA solution of PMDA‐ODA used in this study were purchased from Sigma‐Aldrich. All chemicals and reagents were used as received without further purification.

### Device Fabrication

A patterned 80 nm thick ITO layer (30 Ω square^–1^) on a glass substrate was used as a transparent anode. The ITO substrate was ultrasonically cleaned with ethanol, a detergent, and deionized water and then dried with N_2_ gas. Just before use, the ITO substrate was treated with an ultraviolet ozone cleaner for 5 min. For the coating of a HIL, the aqueous solution of PEDOT:PSS was spin coated onto the ITO substrate and annealed at 120 °C for 1 h to form a 30 nm thick PEDOT:PSS HIL. To form a PAA‐PI interlayer, a diluted precursor solution of PAA in *N*‐methyl‐2‐pyrrolidone (1:20 volume ratio) was spin‐coated onto the PEDOT:PSS HIL at 5000 rpm for 35 s, after which the coated film was pre‐baked at 80 °C for 30 min, yielding a 10 nm thick PAA layer on the PEDOT:PSS HIL. The PAA precursor layer on the substrate was then annealed at 180 °C for 1 h to imidize the PAA precursor layer to the PAA‐PI copolymer interlayer (6 nm). Subsequently, the PAA‐PI copolymer‐coated substrate was transferred into a thermal deposition chamber and, to form the EML, a 45 nm thick layer of m‐MTDATA doped with Ir(ppy)_2_(acac) was then deposited onto the PAA‐PI interlayer via thermal deposition at a base pressure below 2.0 × 10^–6^ Pa. Next, a TmPyPB ETL (≈45–55 nm) and a 0.5 nm thick LiF EIL were evaporated successively. Finally, a 70 nm thick Al cathode and a 60 nm thick Ag capping layer were deposited onto the top of the LiF EIL via thermal evaporation. Thus, the fabricated OLED structure was [ITO anode/PEDOT:PSS HIL/PAA‐PI interlayer/m‐MTDATA:Ir(ppy)_2_(acac) EML/TmPyPB ETL/LiF EIL/Al/Ag cathode]. The light‐emitting active area of the fabricated OLED was 2 × 3 mm^2^ in size. To characterize the single‐carrier transport properties, HODs were fabricated with the device structure of [ITO/PEDOT:PSS HIL/PAA‐PI interlayer/m‐MTDATA:Ir(ppy)_2_(acac)/MoO_3_/Ag], and EODs were also produced with the device structure of [ITO/ZnO/Cs_2_CO_3_/PAA‐PI interlayer/m‐MTDATA:Ir(ppy)_2_(acac)/TmPyPB/LiF/Al].

### Film and Device Characterization

The surface morphology and surface potential of each functional layer were investigated by noncontact AFM and simultaneous KPFM (FlexAFM, Nanosurf Inc.) by applying AC voltage of 1 V at a frequency of 18 kHz to a Pt/Ir‐coated silicon tip. Highly oriented pyrolytic graphite (mosaic spread: 0.8° ± 0.2, Optigraph GmbH) was used to calibrate the surface potentials. To identify the energy levels of the functional layers, UPS (PHI 5000 Versa Probe, ULVAC‐PHI Inc.) was utilized in an ultrahigh‐vacuum chamber at a base pressure of 1 × 10^−6^ Pa, equipped with a He I photon line (h*ν* = 21.22 eV) from a He‐discharge lamp as an excitation source. The optical properties of the functional layers were then investigated using a UV–vis spectrometer (Cary 1E, Varian). The EL performance of the fabricated OLED was measured using a power source meter (2400, Keithley) and a chromameter (CS‐2000, Konica Minolta). A LED measurement system (LED Master‐120, J&C Technology Inc.) with an integrating sphere was also used to measure the emission characteristics and EQEs of the fabricated OLEDs. With ImageJ software, the sizes of the dark spots that formed on the OLEDs were analyzed from their EL images. Device stability measurements were also taken using an OLED Lifetime System from Ossila (T2005B2‐UK) to measure the relative luminance decay of the reference and sample OLEDs.

## Conflict of Interest

The authors declare no conflict of interest.

## Supporting information

Supporting Information

## Data Availability

The data that support the findings of this study are available on request from the corresponding author. The data are not publicly available due to privacy or ethical restrictions.
