# Supplementary material for: Highly Efficient and Stable Organic Light‐Emitting Diodes with Inner Passivating Hole‐Transfer Interlayers of Poly(amic acid)‐Polyimide Copolymer
Source: Adv Sci (Weinh). 2022 Jan 27;9(9):2105851. doi: 10.1002/advs.202105851 (PMC8948599; doi:10.1002/advs.202105851)
Supplement: Supplementary file 1 — Supporting Information [file ADVS-9-2105851-s001.pdf]

ADVANCED SCIENCE
Open Access

Supporting Information

for *Adv. Sci.*, DOI 10.1002/advs.202105851

Highly Efficient and Stable Organic Light-Emitting Diodes with Inner Passivating Hole-Transfer Interlayers of Poly(amic acid)-Polyimide Copolymer

*Jaewoo Park, Wonsun Kim, Yushika Aggawal, Kichul Shin, Eun Ha Choi and Byoungchoo Park**

ADVANCED SCIENCE
Open Access

Supporting Information

for *Adv. Sci.*, DOI: 10.1002/advs.202105851

Highly Efficient and Stable Organic Light-emitting Diodes with Inner Passivating Hole-transfer Interlayers of Poly(amic acid)-polyimide Copolymer

Jaewoo Park[1,2], *Wonsun Kim*[1], *Yushika Aggawal*[1], *Kichul Shin*[1], *Eun Ha Choi*[1,2], *and Byoungchoo Park**[1,2]

Supporting Information

Highly Efficient and Stable Organic Light-emitting Diodes with Inner Passivating Hole-transfer Interlayers of Poly(amic acid)-polyimide Copolymer

Jaewoo Park[1,2]*, Wonsun Kim*[1]*, Yushika Aggawal*[1]*, Kichul Shin*[1]*, Eun Ha Choi*[1,2]*, and Byoungchoo Park**[1,2]

J. Park, W. Kim, Y. Aggawal, Dr. K. Shin, Prof. Dr. E. H. Choi, Prof. Dr. B. Park
[1]Department of Electrical and Biological Physics, Kwangwoon University, Wolgye-Dong, Seoul 01897, South Korea
[2]Department of Plasma-Bio Display, Kwangwoon University, Wolgye-Dong, Seoul, 01897, South Korea
E-mail: bcpark@kw.ac.kr

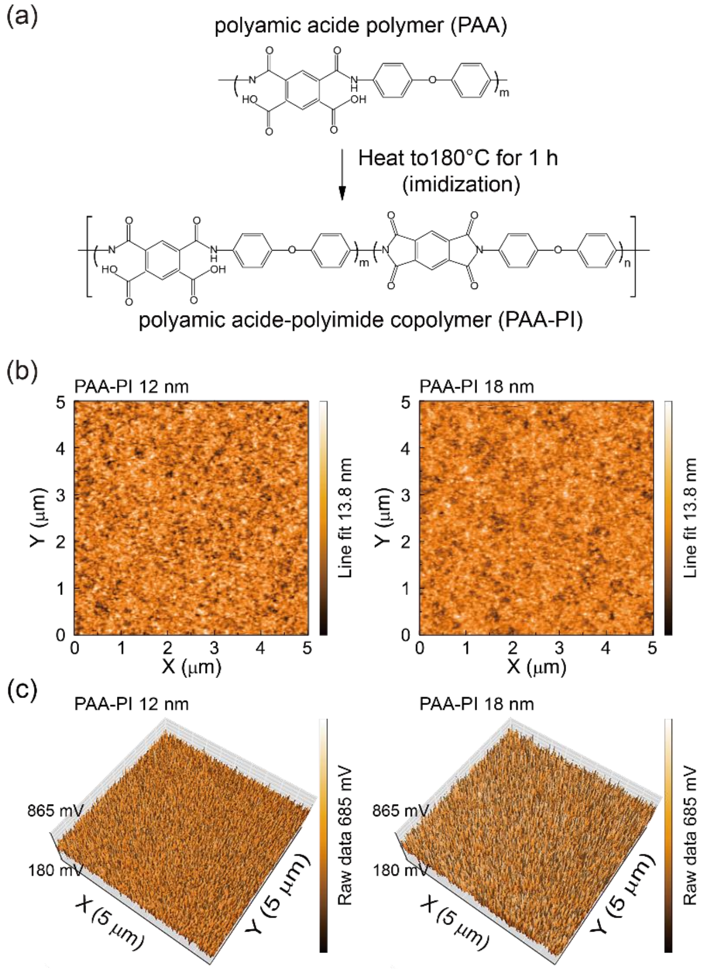

Figure S1. a) Synthetic route to poly(amic acid)-polyimide copolymer PAA-PI. b) AFM topography images and c) corresponding KPFM potential maps of PAA-PI layers with different film thicknesses on ITO/30-nm-thick PEDOT:PSS layers.

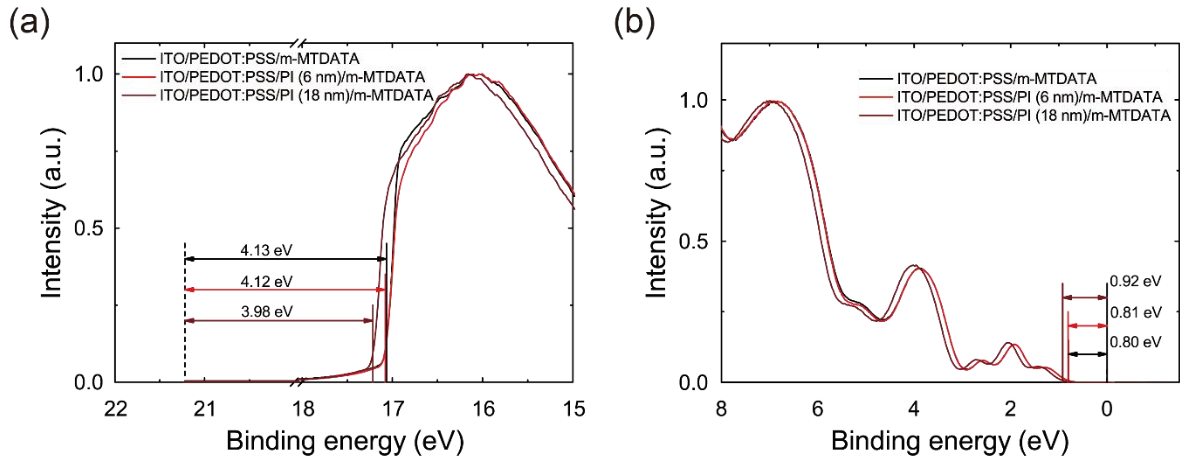

Figure S2. Ultraviolet photoelectron spectroscopy (UPS) spectra of m-MTDATA layers on thin PAA-PI layers with different film thicknesses on ITO/PEDOT:PSS layers for the determination of a) their work functions and b) valence band maximum levels. The work function is given by W = 21.22 – (cutoff energy).

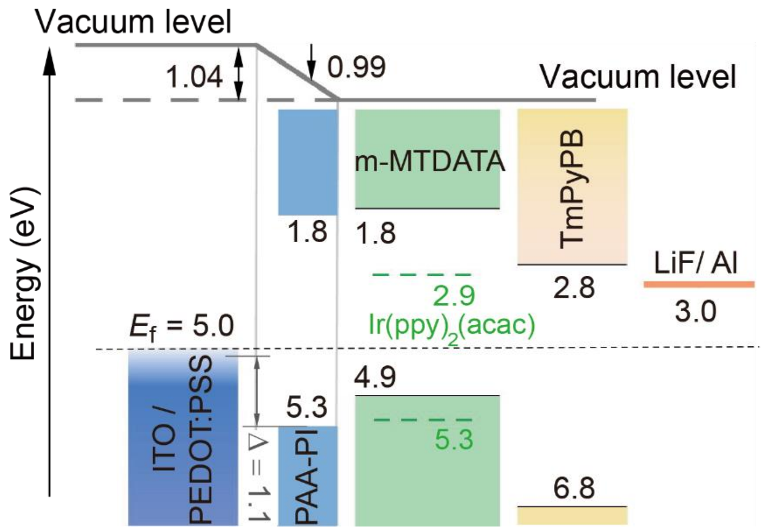

Figure S3. Energy-level diagram of a sample OLED with a PAA-PI interlayer (18 nm). Energy barrier Δ between the PEDOT:PSS HIL and the PAA-PI interlayer (18 nm): Δ = $\Delta E_{F(PEDOT:PSS/PAA\text{-}PI)} - \Delta E_{HOMO(PEDOT:PSS/PAA\text{-}PI)}$ = (5.02 - 4.03) - (5.20 - 5.34) eV ~ 1.13 eV.

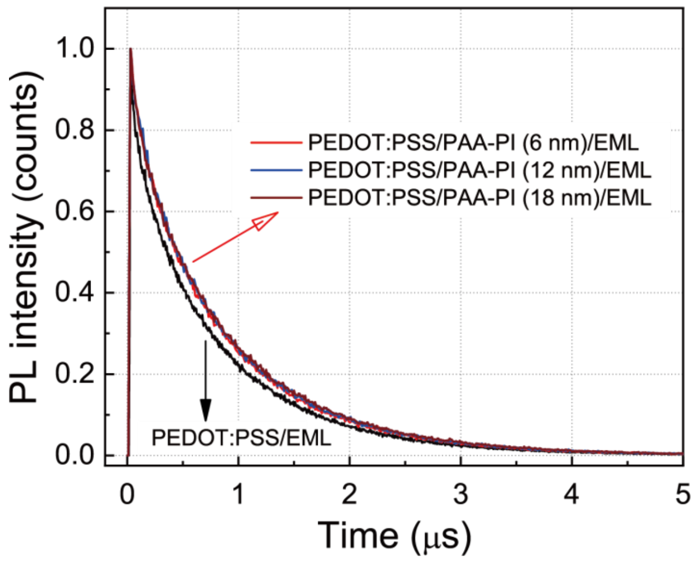

Figure S4. Time-resolved PL signals of the glass/PEDOT:PSS/EML, glass/PEDOT:PSS/PAA-PI (6 nm)/EML, glass/PEDOT:PSS/PAA-PI (12 nm)/EML, and glass/PEDOT:PSS/PAA-PI (18 nm)/EML layers with an EML thickness of 45 nm.

The photoluminescence (PL) decay profiles were measured to observe the exciton quenching of the PEDOT:PSS/EML and PEDOT:PSS/PAA-PI/EML layers. As shown in Figure S4, the exciton lifetimes of the sample layers of PEDOT:PSS/PAA-PI/EML at 522 nm are substantially increased to 0.89-0.92 μs from 0.85 μs for the reference layer of PEDOT:PSS/EML on glass substrates. These increased exciton lifetimes of the PEDOT:PSS/PAA-PI/EML layers clearly indicate the decreases in the exciton quenching effect owing to the existence of the PAA-PI interlayer between the PEDOT:PSS HIL and EML.

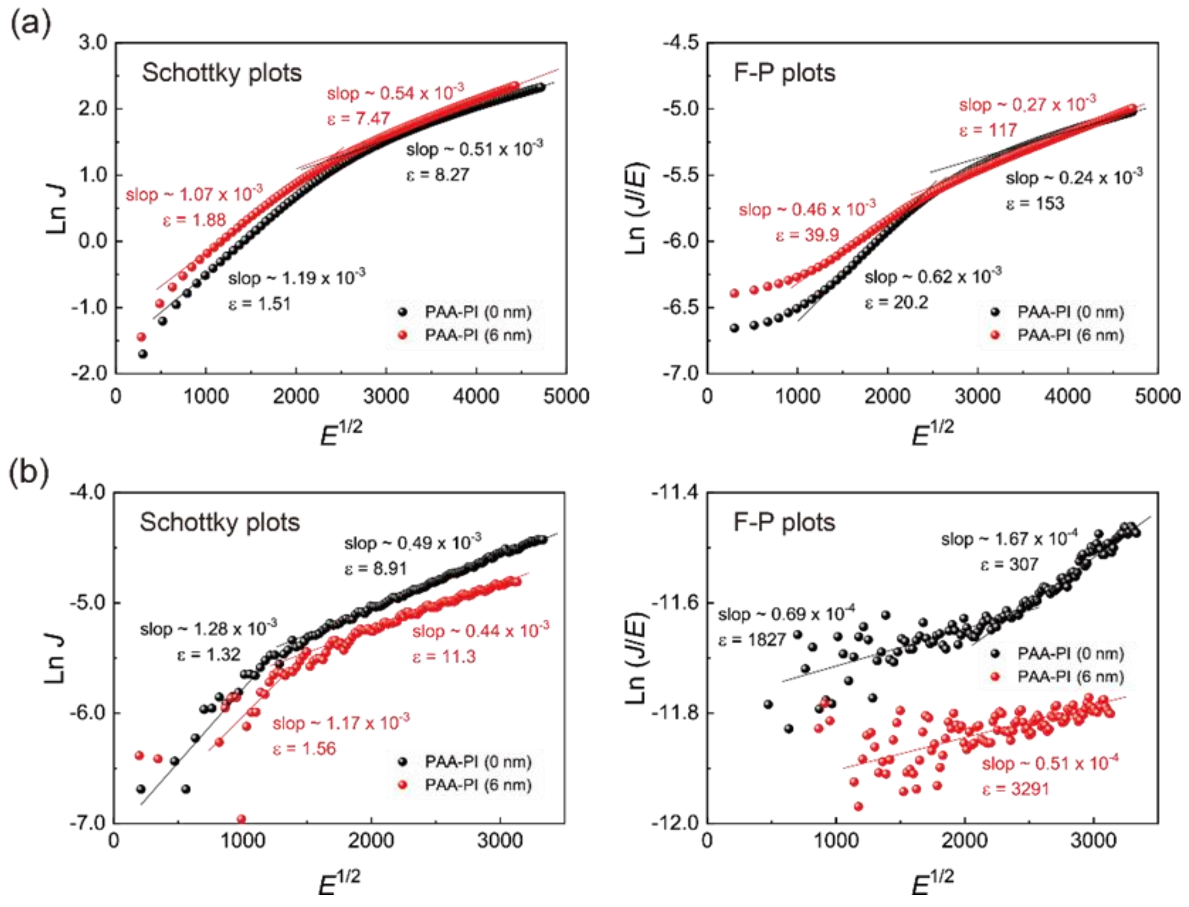

Figure S5. *J*-*V* characteristics of a) hole-only devices and b) electron-only devices without (0 nm) and with a PAA-PI interlayer (6 nm); Left: Schottky plots. Right: F-P plots.

Schottky and Frenkel-Poole (F-P) conduction models are also commonly used to describe interface- and bulk-limited conductions, respectively, under low or moderate electric fields[1-3], together with the Fowler-Nordheim (*F*-*N*) tunneling and Ohmic/space-charge-limited-current (SCLC) conduction models. The relationship of ln(J) versus $E^{1/2}$ should be linear if the interface-limited current conduction is governed by the Schottky model. Similarly, the plot of ln (J/E) versus $E^{1/2}$ would present a linear relationship when the bulk-limited current conduction follows the F-P model.

The panel on the left in Figure S5a shows Schottky plots with ln(J) versus $E^{1/2}$ for both of the HODs studied here. Linear relationships are observed for both cases under low or

moderate electric fields, but the fitted relative dielectric constants (ε_s) are ~1.5 - 1.9 or ~7.5 - 8.3, respectively, which are not reasonable in a comparison with those (~3) in previous reports[4]. Hence, Schottky conduction may exist but is not the main interface-limited conduction mechanism of either HOD.

The panel on the right in Figure S5a shows F-P plots of ln (J/E) versus $E^{1/2}$. The linear relationships shown in these plots suggest that F-P conduction may also exist in the HODs. However, the fitted dielectric constants do not match with the reported values[4]. Hence, F-P conduction is not the main bulk-limited conduction mechanism. Thus, neither Schottky nor F-P conduction defines the type of hole current conduction in either of the HODs studied here.

Next, the panel on the left in Figure S5b shows the Schottky plots of ln(J) versus $E^{1/2}$ for both EODs studied here. Linear relationships are observed but the fitted relative dielectric constants are not feasible in a comparison with reported values. Hence, Schottky conduction may exist but is not the main interface-limited conduction mechanism.

The panel on the right in Figure S5b also shows F-P plots of ln (J/E) versus $E^{1/2}$. The linear relationships shown in these plots also suggest that F-P conduction may exist in the EODs. However, the fitted dielectric constants also do not match the reported values. Hence, F-P conduction is not the main bulk-limited conduction mechanism for either EOD.

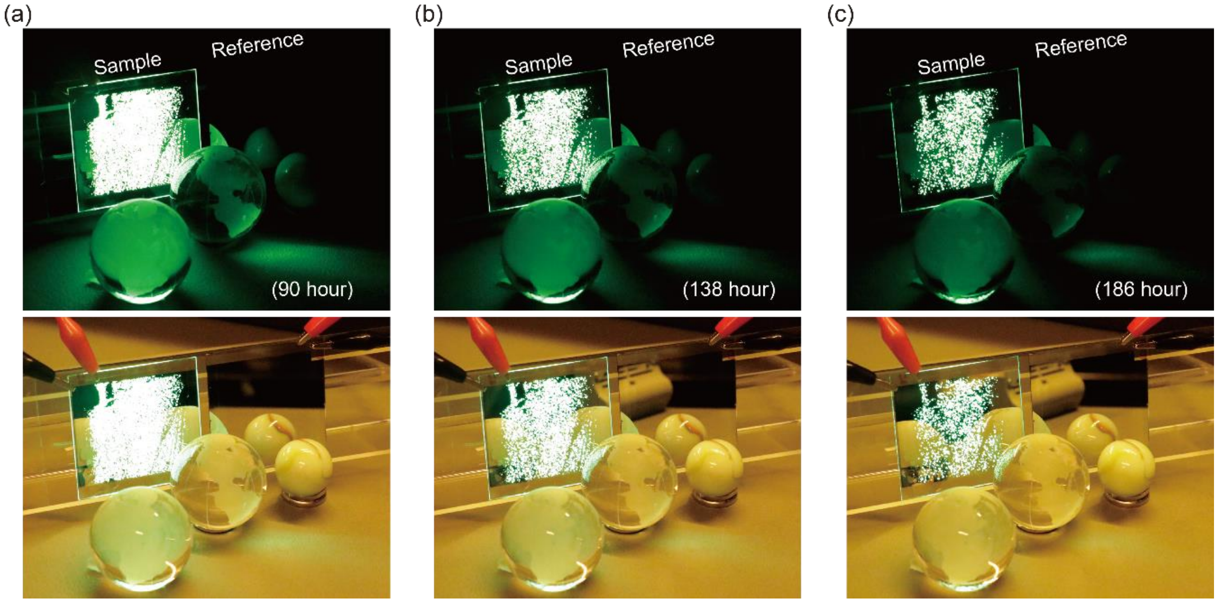

Figure S6. Photographs taken in dark (upper) and bright (lower) ambient light conditions when operating large-area OLED lighting devices (5.5 × 5.5 cm^2, at 6.0 V) without (right, reference) and with (left, sample) a solution-processed 6-nm-thick PAA-PI interlayer a) after 90 h, b) after 138 h, and c) 186 h of storage time in an air atmosphere (23°C, 30%RH, without any external encapsulation), also showing the ease of fabrication of the large-area and stable OLED with the PAA-PI interlayer (an extension of Figure 6 in the manuscript).

References

[1] S. M. Sze, *Physics of Semiconductor Devices*, Wiley Interscience, **1981**.

[2] E. H. Rhoderick, R. H. Williams, *Metal-Semiconductor Contacts 2nd ed.*, Clarendon Press, Oxford, **1988**.

[3] J. Frenkel, *Phys. Rev.* **1938**, *54*, 647.

[4] A. Ligthart, *Phd Thesis*, Universiteit Technische **2020**.
